# The Association of Vitamin A and Vitamin D with Hypertension in Children: A Case-Control Study

**DOI:** 10.1155/2018/9295147

**Published:** 2018-12-06

**Authors:** Xiaohua Liang, Min Chen, Ping Qu, Guang Hao, Yisong Huang, Jie Chen, Tingyu Li

**Affiliations:** ^1^Children's Hospital of Chongqing Medical University, Ministry of Education Key Laboratory of Child Development and Disorders, Key Laboratory of Pediatrics in Chongqing, China International Science and Technology Cooperation Base of Child Development and Critical Disorders, Chongqing, China; ^2^Augusta University, Georgia Prevention Institute, Medical College of Georgia, Augusta, GA, USA

## Abstract

**Background:**

The prevalence of hypertension in children increases rapidly. This paper is to investigate the association of vitamin A and serum 25(OH)D level with hypertension and to explore the risk factors of hypertension in children.

**Methods:**

164 children (age: 6-12 years, females: 49.39%) were included in this case-control study. The serum vitamin A and serum 25(OH)D level, the transcription level of RARs and RXRs, 25(OH)D receptor, and the retinol acyltransferase (LRAT), an indicator of vitamin A storage function, were measured.

**Results:**

The serum vitamin A level in hypertensive subjects was not significantly different compared to control, but the serum 25(OH)D level was significantly lower in hypertensive subjects compared to control (38.22±12.00umol/L vs. 43.28±12.33 umol/L,* P=*0.02). The transcription levels of RAR*α*, RAR*β*, and RAR*γ* were not significantly different between the two groups; but the LRAT was lower in the hypertensive group than that in the control (*P*<0.001). Compared with control group, the level of 25(OH)D receptor was lower in hypertension children (*P*=0.003). Logistic regression model showed that LRAT, HDL, and breastfed duration were negatively associated with blood pressure, and waist circumference was positively associated with blood pressure.

**Conclusions:**

The LRAT, serum 25(OH)D, and 25(OH)D receptor were significantly associated with blood pressure level, and both breastfed and HDL-C were independent protective factors of blood pressure level in children.

## 1. Introduction

Hypertension is a major risk factor for cardiovascular disease and stroke. It is one of the leading causes of death and disability worldwide. The prevalence of hypertension in children ranges from 3%-12.6% [1, 2], and is still increasing in China. Lifestyle interventions are recommended for children with primary hypertension, but pharmacologic treatment is debatable for children with primary hypertension [3]. Moreover, little nutrition intervention study has been done in children with primary hypertension. Therefore, it is pivotal to investigate the influence of nutrition on hypertension in childhood and develop the evidence for potential safe alternative treatment for children with hypertension.

In this study, we aim to explore the risk factors of hypertension in children. All-transretinoic acid (atRA), a biologically active metabolite of vitamin A (VA), has been shown to regulate the gene expression of components of rennin angiotensin system, including renin, angiotensin-converting enzyme, angiotensin II, and its type 1 receptor in animal studies [4]. On the other hand, vitamin D (VD) refers to a group of fat-soluble secosteroids responsible for increasing intestinal absorption of calcium, magnesium, and phosphate and multiple other biological effects. Animal studies have corroborated the strong effect of VD on the Ang II and blood pressure level [5]. Epidemiological studies reported that VD repletion exerted a clinically significant antihypertensive effect in VD deficient patients with type 2 diabetes [6], cardiovascular disease [7], and general population [8]. However, the controversial results remain [9, 10]. Despite these intriguing laboratory findings, evidence from population studies, especially from children, regarding the relation of VA and VD metabolites, with blood pressure remains limited, and one study from Kao KT et al. [11] found that VD deficiency was associated with hypertension in paediatric obesity. Considering the increasing prevalence of hypertension in children, it is important to investigate the potential association of VA and VD with hypertension in children, and to explore the underlying mechanisms. This case-control study was designed to examine the association of VA and VD with hypertension and to explore other risk factors of essential hypertension in children.

## 2. Methods

### 2.1. Study Population and Sample Size

To achieve the power (1-*β*) of 0.8 under the probability of type I error (*α*) of 0.05, with the parameter of VA mean 0.95 umol/L in case group and 1.05 umol/L in control group, standard deviation of 0.22, and using the formula *n* = (*σ*((*z*_1−*α*/2_ + *z*_1−*β*_)/(*μ*_*A*_ − *μ*_*B*_)))^2^, at least 76 samples were needed for case group and control group, respectively. A total of 164 gender and age (9.81±1.62 years) matched subjects were selected from an established cohort of 6102 children aged 6-12 years in 2013 in Chongqing from 6 elementary schools. Inclusion criteria for the hypertensive group were children aged 6 to 12 years diagnosed with hypertension [12], and who were not under treatment for VA deficiency or VD deficiency and who were not taking antihypertensive drugs. Children with diseases (e.g., diabetes, cardiovascular disease, or cancer) and taking medications, which affect VA and VD absorption and secondary hypertension was excluded from this study. The inclusion criteria for the nonhypertensive group were children without hypertension or any other disease that affect VA and VD absorption and metabolism. All work in this study was conducted in accordance with the ethical guidelines of 1964 Declaration of Helsinki and its later amendments. This study was approved by the Institutional Review Board of Chongqing Medical University and written informed consent was obtained from each child and from parents or guardians prior to their inclusion in the study.

### 2.2. Demographic Variables and Dietary Intake

Demographic and socioeconomic status and medical information were collected by trained nurses and study coordinators through a comprehensive questionnaire. Demographic information included gender, age, family status, birth weight, breastfed, pregnancy hypertension, and gestational diabetes. Socioeconomic status (SES) was represented by father's occupation and father's education level, and the household income and family disease history of hypertension were also investigated.

A semi-quantitative food frequency questionnaire was used to collect the dietary survey. Food was divided into 15 categories, including grains, vegetables, fruits, meat, poultry, fish and shellfish, eggs, milk and dairy products, beans and bean products, nuts, algae, cooking oil, pickles, nutritional supplements, and beverages. Cooking oil consumption was measured in a family unit, through collecting the frequency of eating at home for the family member and the total cooking oil consumption during a period, and then the average intake of cooking oil per day was estimated. The amount of food was investigated by interviewing the parents about the intake amount and the frequency during an interval, and then the total amount of food was averaged to the daily intake. The standardized food containing utensils (bowl, plate, cup and spoon, etc.) were used to display the weight of food to ensure the validity of survey.

### 2.3. Physical Examination

Anthropometric measurements and other physical examinations were conduct by well-trained nurses. Waist circumference was measured twice at the center of the umbilicus over one T-shirt and the values were averaged. Height and weight were also measured by using mobile medical ultrasonic machine (the model is WS-H300D), and body mass index (BMI) was calculated as weight divided by squared height (kg/m^2^), as a measure of general adiposity, and the Z-score of BMI was calculated using the LMS parameters and formula from CDC [13]; waist circumference was used as an alternative measure of central adiposity.

Blood pressure (BP) and heart rate (HR) were measured on three separate occasions with participant in the sit-down position by the OMRON arm type electronic sphygmomanometer (HEM7051) using an appropriately sized BP cuff placed on the right arm. BP measurements were taken at 11, 13, and 15 minutes during a 15-minute relaxation period in one occasion [14]. The average value of all three blood pressure measurements was used to represent resting systolic blood pressure (SBP) and diastolic blood pressure (DBP) level. If the first blood pressure screening met the criteria of hypertension, the second and third measurements were conducted in the following weeks. Subjects were diagnosed with hypertension if all the three measurements of blood pressure met the criteria for hypertension [15]. To rule out secondary hypertension effects, medical history was interviewed, and physical examination was taken on hypertensive subjects.

### 2.4. Biochemical Index

The venous blood (3ml) was drawn in the morning at least 12 hours after fasting and 24 hours after withholding from a high-fat diet and spicy diet for each subject. The serum was separated by 2500 rpm centrifugation at 4°C for 10 min. The automatic biochemical analyzer was used to measure the serum total cholesterol, high density lipoprotein cholesterol (HDL-C), triglyceride, low density lipoprotein cholesterol (LDL-C), and fasting blood glucose (FBG). The VA was measured by high performance liquid chromatography (HPLC) with dissolved mobile phase (which is made up of 97% methanol and 3% water). Anhydrous ethanol and n-hexane were used to extract retinol and dried by nitrogen. VD was also measured by HPLC, after extracting and filtering through acetonitrile and chromatographic column.

### 2.5. RNA Isolation and Analysis

The total RNA isolated from peripheral blood leukocyte was used for real-time polymerase chain reaction (PCR). A 500-ng sample of the total RNA was reverse-transcribed into cDNA using the PrimeScript® RT reagent kit (Takara, Japan) according to the protocol of manufacturer. Retinol acyltransferase (LRAT) was an indicator of vitamin A storage function. The primer sequences for human retinoic acid receptor alpha (RAR*α*), retinoic acid receptor beta (RAR*β*), retinoic acid receptor *γ*(RAR*γ*), LRAT, vitamin D receptor (VDR), and *β*-actin were provided in Supplementary Table 1. The target genes were amplified via real-time PCR using a CFX Connect Real-Time PCR thermocycler (BioRad, USA) and SYBR II Premix Ex TaqTM (Takara, Japan). The PCR was performed according to the following protocol: 95°C for 30 s followed by 40 cycles of 95°C for 5 s and 60°C for 30 s. The melting curve was analyzed, and a specific standard curve was generated for each gene in parallel. Each sample was quantified in triplicate. The gene expression results were calculated by normalizing the concentration of the target gene to that of the endogenous levels of *β*-actin using CFX Manager Software.

### 2.6. Diagnostic Criteria

The diagnostic criteria of hypertension used in this study were according to Mi Jie [12], which is suitable for the growth characteristics of children and teenagers in China. Hypertension was defined as average clinic measured SBP and/or DBP ≥95th percentile (based on age, gender, and height percentiles). Because the oscillometric devices can serve better in the developing children (2-12 years) [16], electronic sphygmomanometer was used to measure blood pressure in this study.

The sufficient VA level was defined as a serum retinol level no less than 1.05*μ*mol/L per milliliter. The relative insufficient VA level was defined as a serum retinol level between 0.70*μ*mol/L and 1.05*μ*mol/L. The VA deficiency was defined as a serum retinol level less than 0.70*μ*mol/L per milliliter [17]. VD deficiency was defined as a 25(OH)D level of less than 50nmol per liter [18].

### 2.7. Statistical Analysis

The continuous variables were reported as means and standard deviations (SD) if the variables meet normal distribution, and t-test was used to test the significance of difference between the two groups. Continuous variables that do not satisfied the normal distribution were expressed as X_50%_ (X_25%_, X_75%_), and the Wilcoxon rank sum test was used for comparison between the two groups. The categorical variables were reported as numbers (n) and percentages of the total (%), and *χ*^2^ test was used to test the difference between two groups. Covariance analysis was used to adjust covariables, when comparing the concentration of serum retinol, 25(OH)D, and VDR between the two groups. Moreover, the correlation coefficients of blood pressure with LRAT, 25(OH)D, and VDR were analyzed by correlation analysis. Multivariate logistic regression model was used to identify the associations of hypertension and risk factors of VA and VD. Multivariate logistic regression model was used to identify the associations of hypertension and risk factors of VA and VD.

The data analysis for this study was generated using SAS 9.4 software (Copyright © 2016 SAS Institute Inc. Cary, NC, USA). The t test and correlation analysis of VA, VD, and PCR in two groups were performed by Graph Pad Prism5 statistical software. Significant difference was determined at the *α* level of 0.05.

## 3. Results

The general characteristics of subjects were presented in Table 1. The mean age was 9.81±1.62 years, and 49.39% were females. There is no significant difference in age, gender, father's occupation, and father's education level between the hypertensive and control group. There is no significant difference in average birth weight between the two groups (3373.5±489.0g vs. 3284.2±478.9g, P=0.24). The rate of being breastfed also showed no significant difference (80.00% vs. 89.29%, P=0.09), but the breastfed duration in hypertensive children was significantly shorter than that in the control group ((7.11±5.08 vs. 8.69±4.93 months,* P*<0.05) (Table 1).

Compared with the control group, BMI, LDL-C, and triglyceride (TRIG) were significantly higher in children with hypertension, but HDL-C was significantly lower in children with hypertension (all* P*<0.01) (Table 1). The total cholesterol and fasting blood glucose (FBG) were not significantly different between the two groups (Table 1).

In Supplementary Table 2 major dietary categories were compared, and there was no obvious difference in major dietary intake, except that the average intakes of nuts, mushrooms, and algae food in the hypertension group were lower than those in the control group (all P<0.05).

**Figure 1:**
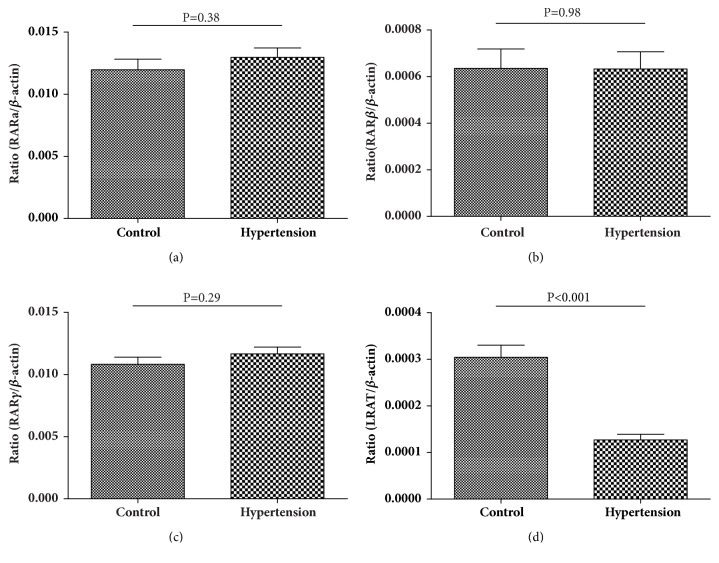
The level of RAR*α*, RAR*β*, RAR*γ* and LRAT mRNA expression (n=164). (a) The level of RAR*α* mRNA expression (Ratio (RAR*α*/*β*-actin)) between the hypertension and control groups, P=0.38. (b) The level of RAR*β* mRNA expression (Ratio (RAR*β*/*β*-actin)) between the hypertension and control groups, P=0.98. (c) The level of RAR*γ* mRNA expression (Ratio (RAR*γ*/*β*-actin)) between the hypertension and control groups, P=0.29. (d) The level of LRAT mRNA expression (Ratio (LRAT /*β*-actin)) between the hypertension and control groups, P<0.001.

**Figure 2:**
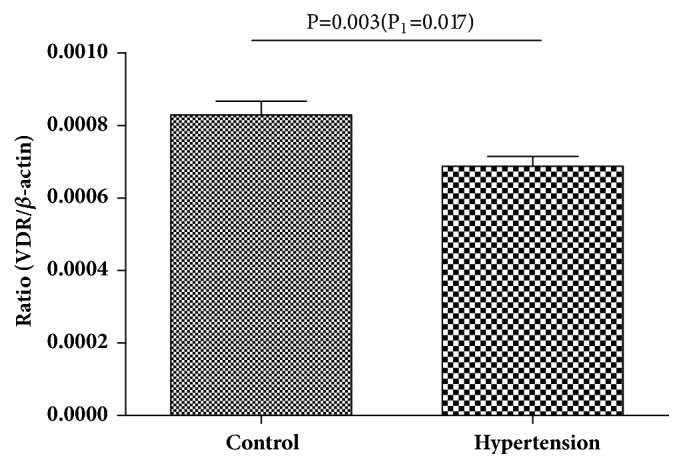
The level of VDR mRNA expression (Ratio (VDR/*β*-actin)) in hypertension and control group, VDR mRNA expression level was lower in children with hypertension than that in control.

**Figure 3:**
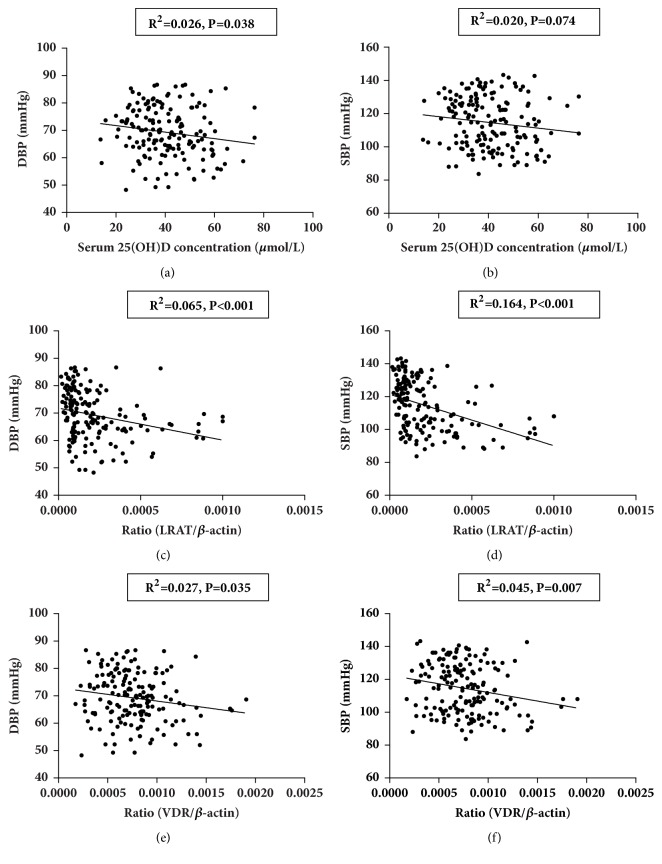
The Correlation Analysis of VA and VD with SBP and DBP (n=164). (a) The correlation between serum 25(OH)D levels (umol/L) and DBP. (b) The correlation between serum 25(OH)D levels (umol/L) and SBP. (c) The correlation between the level of LRAT mRNA expression and DBP. (d) The correlation between the level of LRAT mRNA expression and SBP. (e) The correlation between the level of VDR mRNA expression and DBP. (f) The correlation between the level of VDR mRNA expression and SBP.

After adjusting for age, gender, BMI, WC, TRIG, HDL-C, and LDL-C, the concentration of serum retinol and VA deficiency rate between the two groups has no significant difference (P=0.56 and P=0.12) (Table 1). However, serum 25(OH)D in children with hypertension was lower than that in the control group (38.22±12.00umol/L vs. 43.28±12.33umol/L, P=0.02) (Table 1), whereas the difference of 25(OH)D level between the two groups was reduced after adjusted BMI(P=0.19) and the deficiency rate of 25(OH)D was boundary significant (P=0.06) (Table 1). The results of qPCR showed that there were no significant differences in the mRNA expression level of RAR*α*, RAR*β*, and RAR*γ* between the two groups (Figure 1). The expression level of LRAT in the hypertensive group was significantly lower than that in the control group (Figure 1(d)). The results in Figure 2 showed that the 25(OH)D receptor (VDR) mRNA expression in hypertension group was lower than that in control group(P=0.003), even after adjusted BMI (P=0.017). In Figure 3 the blood pressure negatively associated with LRAT (Figures 3(c) and 3(d)), but not with RAR*α*, RAR*β*, or RAR*γ*. Serum 25(OH) D was significantly correlated with DBP (Figure 3(a)). Both the correlations between the levels of VDR mRNA expression with SBP and DBP were significant in Figures 3(f) and 3(e) (all P<0.05).

The association of LRAT and VDR with BP was analyzed in multivariable logistic regression model in Table 2, and the covariates of Z-score of BMI, breastfed, WC, HDL-C, LDL-C, LRAT, VDR, nuts, mushrooms, and algae food were included. The serum levels of VA and LRAT are highly correlated, as well as the serum level of VD and VDR. Hence, just LRAT and VDR were included in the final model. As shown in Table 2, the WC were positively associated with the BP, but breastfed, LRAT, and HDL-C were negatively associated with BP. The correlation analysis of VDR and LRAT mRNA expression level showed that LRAT increased according to VDR level in children with hypertension (R=0.24, P=0.03), but that was not observed in control group (R=-0.03, P=0.82).

## 4. Discussion

This study demonstrated that serum LRAT strongly correlated with blood pressure in children, and the serum 25(OH)D and its receptor VDR in children with hypertension were lower than those in the control group. The results showed that longer breastfed duration and higher HDL-C were the protective factors of hypertension, and abdominal obesity was associated with increased risk of hypertension in multivariable logistic regression model.

LRAT level was negatively associated with blood pressure in this study. LRAT was a key enzyme in the transformation of retinol and retinyl esters and storage of serum retinol in the tissues [19]. LRAT was lower in hypertensive group than that of control in univariate and multivariate regression model which adjusted breastfed, BMI, WC, and lipid, suggesting that LRAT storage was disorder in children with hypertension. Study from rat revealed that all-transretinoic acid supplement can increase gene and protein expressions of ACE2, resulting in the reduction of blood pressure [4], and all-transretinoic acid also has a significant inhibitory effect on cardiac remodeling, by inhibiting the expression of renin-angiotensin system [20, 21]. However, in this childhood hypertension study, the results have shown that the expressions of RARs (*α*, *β*, and *γ*) in the peripheral blood have no significant difference between the two groups, which may be related to the delayed response of RARs, as retinoic acid receptor showed over expression when serum retinol was deficiency in the tissue. Whether there is VA deficiency in tissue in hypertensive children has not been reported yet, but study has shown that obesity, an important risk factor for hypertension, leads to functional VA deficiency in many organs with decreased expression of stellate cells markers (LRAT in liver; vimentin in pancreas and kidney) [22].

The results also showed that the serum 25(OH)D and its receptor VDR had negative relationship with blood pressure. Serum 25(OH)D and VDR in children with hypertension were lower than those in the control group in univariate and multivariate regression analyses. Moreover, 25(OH)D deficiency was inversely associated with hypertension level, indicating that vitamin D depletion exacerbates hypertension by influencing VDR [23, 24]. This result is consistent with the cohort study in adults which found an inverse association between plasma 25(OH)D and hypertension [8, 25].

LRAT expression level increased accompanying with the increase of VDR expression level in children with hypertension, suggesting VA associated with 25(OH)D in the blood pressure regulation process which need further research in future. Besides, HDL-C was negatively associated with blood pressure, consistent with other studies that show that higher HDL-C was likely to induce lower blood pressure level [26], and HDL-C promotes vasodilatation via its effect on endothelial nitric oxide syntheses [27]. Results found that there are underlined pathogenetic factors and pathophysiological mechanisms linking obesity to hypertension [28, 29]. In our study, WC in children with hypertensive was higher than that of the control and the result agreed with study from Grober-Gratz D. et al. [27] and WC is associated with increased SBP [30]. Another interesting observation was that shorter breastfed duration was associated with worsening blood pressure in this study, and these findings are consistent with other studies both from childhood and adulthood [31–33], suggesting that advocate prolong breastfed duration is helpful to protect cardiology disease in later life. The results also found that greater WC were associated with increased risk of hypertension, indicating that childhood obesity would increase the risk of hypertension and developing a cardiovascular disease in adulthood [34].

It is the first time that the negative relationship of LRAT and VDR with childhood hypertension was found, suggesting that supplement of VA and VD in children with hypertension may play important roles in cardiovascular protection. However, this study also has some limits. Because we did not conduct a prospective cohort study to explore the damage extent of VA and VD deficiency to the target organ of hypertension, it could not verify etiological hypothesis of VA and VD on children hypertension.

## 5. Conclusions

Among children aged 6-12 years, serum LRAT, serum 25(OH)D, and VDR level were negatively associated with blood pressure level. These results suggest that improvement of VA and VD nutrition supply may be a potential usefulness intervention as hypotheses for further study. In addition, we found that longer breastfed duration was associated with decrease blood pressure level, suggesting that advocate breastfed was a potential method to control blood pressure level even in childhood. Besides, higher WC were related to deteriorated blood pressure level in children, suggesting through increasing exercise and healthy dietary to control obesity were valid methods to control blood pressure from childhood.

## Figures and Tables

**Table 1:** Demographic information in the hypertensive group and the control group.

Variables	Total	Hypertension	Control	P
N	164	80	84	
Age, years	9.81(1.62)	9.74(1.77)	9.89 (1.45)	0.56
Gender, n (%)				
Male	83(50.61%)	42(52.50%)	41(48.81%)	0.64
Female	81(49.39%)	38(47.50%)	43(51.19%)	
**Pregnancy and neonatal variables**				
Birth weight, g	3327.74 (484.43)	3373.50(489.0)	3284.20(478.90)	0.24
Breastfed, n (%)				
Yes	139(84.76%)	64(80.00%)	75(89.29%)	0.09
No	25(15.24%)	16(20.00%)	9(10.71%)	
Duration of breastfed, month	7.92(5.05)	7.11 (5.08)	8.69(4.93)	0.04
PIH				
Yes	5(3.04%)	2 (2.50%)	3(3.57%)	0.18
No	159(96.96%)	78 (97.50%)	81(96.43%)	
**Parents' education level**				
<9	95(57.93%)	44(55.00%)	51(60.71%)	0.34
~15	54(32.93%)	26(32.50%)	28(33.33%)	
>15	15(9.15%)	10(12.50%)	5(5.95%)	
**Anthropometric variables, mean(SD)**				
SBP, mmHg	114.45(9.04)	127.21(7.87)	101.53(7.69)	<0.01
DBP, mmHg	69.13(9.90)	75.82(8.43)	62.36(5.87)	<0.01
Waist, cm	62.13(5.91)	65.16(5.68)	55.09(5.64)	<0.01
BMI, kg/m^2^	19.45(4.59)	22.51(4.39)	16.36(1.94)	<0.01
**Serum indexes, mean(SD) **				
Chol, mmol/L	4.03(0.63)	4.00(0.68)	4.06(0.59)	0.77
FBG, mmol/L	4.96(0.53)	4.93(0.62)	4.99(0.41)	0.39
HDL-C, mmol/L	1.26(0.44)	1.11(0.31)	1.40(0.49)	<0.01
LDL-C, mmol/L	2.31(0.59)	2.40(0.66)	2.22(0.50)	<0.01
Trig, mmol/L	1.12(0.60)	1.29(0.61)	0.95(0.54)	<0.01
Serum VA^*∗*^, umol/L	0.88(0.23)	0.89(0.23)	0.87(0.22)	0.56
25(OH)D, umol/L	40.81(12.34)	38.22(12.00)	43.28(12.33)	0.02
25(OH)D^#^, umol/L	40.81(12.34)	39.10(12.00)	42.44(12.33)	0.19
**VA deficiency, n(**%**) **	35(21.34%)	13(16.25%)	22(26.19%)	0.12
**VD deficiency, n(**%**)**	125(76.22%)	66(82.50%)	59(70.24%)	0.06

PIH: pregnancy induced hypertension, SBP: systolic blood pressure, DBP: diastolic blood pressure,  ^*∗*^adjusted age, gender, BMI, WC, TRIG, HDL-C, and LDL-C, and  ^#^adjusted BMI.

**Table 2:** Logistic regression model analyzed the risk factors of children with hypertension.

**Variables**	**β**	***SE***	***OR***	**95**%*CI*	**Wald ** *χ* ^*2*^	***P***
Nuts, g	0.012	0.010	1.012	(0.993, 1.031)	1.565	0.211
Mushrooms and algae, g	0.005	0.012	1.005	(0.981, 1.029)	0.140	0.709
Z-score of BMI	-0.601	0.340	0.548	(0.282, 1.067)	3.133	0.077
Breastfed, month	0.163	0.060	1.177	(1.047, 1.324)	7.482	0.006
Waist, cm	-0.107	0.035	0.898	(0.839, 0.962)	9.547	0.002
Triglyceride, mmol	-0.293	0.517	0.746	(0.271, 2.056)	0.321	0.571
LDL-C, mmol	-0.115	0.536	0.891	(0.312, 2.55)	0.046	0.830
HDL-C, mmol	1.764	0.861	5.833	(1.08, 31.504)	4.199	0.040
LRAT	0.101	0.025	1.106	(1.054, 1.161)	16.749	<.0001
VDR	0.114	0.079	1.120	(0.96, 1.308)	2.068	0.150

## Data Availability

The data used to support the findings of this study are included in the article. Requests for access to these details and data should be made to Xiaohua Liang (Email: liangxiaohua666@sina.com).

## Supplementary Materials

Supplementary Materials STable 1. Primer orders. STable 2. Dietary intakes, Median (P25, P75), g.
