# Supplementary material for: The Association of Vitamin A and Vitamin D with Hypertension in Children: A Case-Control Study
Source: Int J Hypertens. 2018 Dec 6;2018:9295147. doi: 10.1155/2018/9295147 (PMC6304547; doi:10.1155/2018/9295147)
Supplement: Supplementary Materials — STable 1. Primer orders. STable 2. Dietary intakes, Median (P25, P75), g. [file 9295147.f1.zip › Supplement table 1.doc_IJHY_2580577.doc]

**Supplement table 1.** Primer orders

| Primers | Orders |
| --- | --- |
| Human-RARα forward | TCCGCCGCAGCATCCAGAAGAACAT |
| Human-RARα reverse | ACTCGGGCTTGGGCACCTCCTTCTT |
| Human-RARβ forward | GGAACACAGCAGAGCACAG |
| Human-RARβ reverse | ACGAGTGGTGACTGACTGAC |
| Human-RARγ forward | CGCCGAAGCATCCAGAAGAAC |
| Human-RARγ reverse | GCGATTCCTGGTCACCTTGTTG |
| Human-LRAT forward | GGAGGACTTCGCCTACGGA |
| Human-LRAT reverse | TTGTCGGACTGGGGACTGAT |
| Human-VDR forward | GTCAGTTACAGCATCCAAAAGG |
| Human-VDR reverse | GACTCATTGGAGCGCAACATG |
| Human-β-actin forward | GTGAAGGTGACAGCAGTCGGTT |
| Human-β-actin reverse | GAGAAGTGGGGTGGCTTTTAGGA |
