# Supplementary material for: The Association of Vitamin A and Vitamin D with Hypertension in Children: A Case-Control Study
Source: Int J Hypertens. 2018 Dec 6;2018:9295147. doi: 10.1155/2018/9295147 (PMC6304547; doi:10.1155/2018/9295147)
Supplement: Supplementary Materials — STable 1. Primer orders. STable 2. Dietary intakes, Median (P25, P75), g. [file 9295147.f1.zip › Supplement table 2.doc_IJHY_2580579.doc]

| Supplement table 2. Dietary intakes, Median (P25, P75), g | | | | |
| --- | --- | --- | --- | --- |
| **Variables** | **Total** | **Hypertension** | **Control** | P |
| Cereals and potatoes | 190.0(107.1,250.0) | 190.0(100.0,250.0) | 190.0(150.0,250.0) | 0.28 |
| Vegetables | 200.0(100.0,250.0) | 200.0(100.0,250.0) | 200.0(107.1,250.0) | 0.74 |
| Fruit | 150.0(71.4,250.0) | 150.0(75.0,232.1) | 195.0(71.4,267.9) | 0.14 |
| Red meat | 100.0(50.0,150.0) | 100.0(50.0,150.0) | 100.0(80.9,150.0) | 0.09 |
| Poultry | 50.0(16.7,71.4) | 42.9(15.5,57.1) | 50.0(16. 7,71.4) | 0.54 |
| Fish | 34.0(14.3,34.0) | 34.0(14.3,34.0) | 34.0(14.3,35.7) | 0.68 |
| Eggs | 50.0(21.4,100.0) | 50.0(21.4,78.6) | 50.0(21.4,100.0) | 0.93 |
| Milk | 200.0(49.1,250.0) | 245.0(42.0,250.0) | 200.0(71.4,250.0) | 0.34 |
| Bean food | 21.4(9.9,58.0) | 21.4 (7.1,50.0) | 21.4 (14.3,96.4) | 0.13 |
| Nuts | 14.3(1.7,28.6) | 5.8 (0.8,22.4) | 16. 7(5.1,35.7) | <0.01 |
| Mushrooms and algae food | 7.1(0.1,26.0) | 3.4 (0,14.6) | 14.3(1.5,35.7) | <0.01 |
| Oils | 50.0(30.0,100.0) | 50.0(30.0,100.0) | 50.0(30.0,100.0) | 0.93 |
| Pickle | 26.0(7.1,26.0) | 26.0(8.3,26.0) | 21.4 (7.1,26.0) | 0.43 |
| Nutritional supplements | 0(0,3.4) | 0(0,1.4) | 0(0,7.1) | 0.65 |
| Beverage | 11.7(0,62.9) | 15.5(0,71.4) | 7.7(0,35.7) | 0.59 |
